# Fasting and postprandial Ghrelin levels in Parkinson’s disease: a systematic review and meta-analysis

**DOI:** 10.1038/s41531-025-01066-0

**Published:** 2025-07-14

**Authors:** Henrique José Cavalcanti Bezerra Gouveia, Osmar Henrique dos Santos-Júnior, Johannes Frasnelli

**Affiliations:** 1 Studies in Nutrition and Phenotypic Plasticity Unit, Center for Health Sciences, Federal University of Pernambuco, Recife-Pernambuco, Brazil; 2 Graduate Program in Neuropsychiatry and Behavioral Sciences, Center for Medical Sciences, Federal University of Pernambuco, Recife-Pernambuco, Brazil; 3 Department of Anatomy, Université du Québec à Trois-Rivières, Trois-Rivières, QC Canada; 4 Research Centre, Sacré-Coeur Hospital Montréal, Montréal, QC Canada; 5 Research Center, Institut universitaire de gériatrie de Montréal, Montréal, QC Canada

**Keywords:** Olfactory system, Parkinson's disease, Neurophysiology

## Abstract

Parkinson’s disease (PD) is a progressive movement disorder with motor and non-motor symptoms, including gastrointestinal and olfactory dysfunctions. These symptoms may be associated with plasma concentrations of the hormone ghrelin. Lower fasting and postprandial plasma levels of total and active ghrelin were reported in PD, despite heterogeneous findings. In this meta-analysis, we assessed the magnitude of ghrelin dysregulation in PD and explored associated factors. We included quasi-experimental and observational studies assessing fasting and postprandial plasma concentrations of total and/or active ghrelin in individuals with PD and controls (eight studies; 985 subjects). Compared to controls, fasting individuals with PD exhibited a significant reduction in total and active ghrelin concentrations. PD also showed significantly reduced postprandial concentrations of total and active ghrelin. This meta-analysis suggests that ghrelin may be crucially involved in the dysfunctions often observed in PD. Further studies should explore factors such as sex, drug therapy, and disease stages.

## Introduction

Parkinson’s disease (PD) is one of the fastest-growing neurological conditions in the world^1^. It is characterized as a progressive movement disorder that includes bradykinesia, rigidity, and tremor at rest, as well as non-motor symptoms that affect sensory perception, cognition, mood, motivation, sleep, and autonomic functions^2,3^. Age is the main risk factor, but other factors such as sex and environmental factors are associated with the risk of developing PD^4–6^. PD is pathologically determined by the aggregation of alpha-synuclein fibers, known as Lewy bodies, which progress from the enteric nervous system and the olfactory bulb to the central nervous system, especially the substantia nigra (SN), via neuroanatomical connections^7^. Thus, gastrointestinal and olfactory dysfunctions are considered the most common non-motor symptoms in individuals with PD, present in approximately 80-90% of patients, respectively, being present in the early stages and progressing as the condition evolves^8–10^.

Although the exact neurobiological underpinnings of gastrointestinal and olfactory alterations are unclear, they may be related to the secretion of gastrointestinal peptides, and particularly ghrelin. Ghrelin’s role in the gastrointestinal system is closely related to its gastric production. Primarily secreted by the oxyntic glands in the gastric fundus, ghrelin is a hormone that stimulates hunger and eating by acting on the brain, in areas such as the hypothalamus and the nucleus of the solitary tract, which are mainly responsible for the homeostatic control of eating behavior and energy balance^11^. Ghrelin levels increase before eating and are rapidly reduced afterwards^12,13^. This is essential for gut motility and mucosal protection, and an alteration in ghrelin expression is associated with functional gastrointestinal disorders like functional dyspepsia and irritable bowel syndrome (IBS)^14^. Recent evidence from PD transgenic mice also supports that alterations in the dorsal motor nucleus of the vagus nerve (DMV), particularly the loss of choline acetyltransferase (ChAT)-positive neurons, play an important role in the GI disturbances observed in PD^15^. The damage to these neurons caused a rapid reduction in total and active plasma ghrelin levels, while chemogenetic activation reversed this condition^15^.

Ghrelin’s actions extend beyond the gastrointestinal system and include the central nervous system. It modulates the hedonic control of eating behavior, in the systems involved in learning and motivation, mainly in the ventral tegmental area (VTA), the striatum, and the hippocampus, influencing reactivity to food^16^. Further, ghrelin influences olfaction, possibly via the presence of receptors in different cells of the olfactory bulb, resulting in increased olfactory sensitivity and exploratory sniffing^17,18^.

As the only orexigenic hormone, ghrelin is part of the molecular regulatory interface between energy metabolism and neuroendocrine processes. This axis appears to be particularly affected in neurodegenerative conditions such as Alzheimer’s, Parkinson’s, and Huntington’s diseases^19^. Ghrelin plays a neuroprotective role by acting as a survival factor for dopaminergic neurons, modulating microglial activation, preserving mitochondrial function, regulating cytokine and apoptotic pathways, and enhancing the activity of antioxidant enzymes. It inhibits microglial activation by suppressing matrix metalloproteinase-3 (MMP-3) expression in dopaminergic neurons, while its effects on mitochondrial function are mediated by the activation of uncoupling protein-2 (UCP2), which reduces 1-methyl-4-phenyl-1,2,5,6 tetrahydropyridine (MPTP)-induced loss of nigral dopaminergic cells^20,21^. Pretreatment with ghrelin prior to MPTP exposure prevented downregulation of Bcl-2 and upregulation of Bax^22,23^, as well as reducing caspase-3 activation in the SN^22^. In A53T mice, which have a mutation that causes early-onset and rapidly progressing Parkinsonism, ghrelin treatment resulted in a reduction in interleukin-6 and an increase in superoxide dismutase 1 (SOD1)^23^.

Furthermore, a recent study indicated a marked reduction in ghrelin receptor (GHSR) regulation in dopaminergic neurons derived from patients with parkin gene (PARK2) mutations^24^. Inhibition of GHSRs in the substantia nigra pars compacta (SNpc) caused motor deficits, supporting the role of ghrelin signaling in the pathophysiology of PD^24^. Given that the classic motor symptoms of PD are caused by neuronal loss in the SNpc and by dopamine depletion in the striatum^25^, the concentration of ghrelin may be a potential early peripheral biomarker for PD^19^.

Lower fasting and postprandial plasma levels of both total and active ghrelin have been reported in PD^26^, which may consequently be associated with olfactory and gastrointestinal dysfunctions, and changes in eating patterns and nutritional status associated with various changes in homeostatic and hedonic eating behavior^11,14,16,27,28^. Total ghrelin represents the levels of deacylated and acylated ghrelin. To activate its only known receptor, ghrelin requires acylation, i.e., the binding of a fatty acid side chain to its serine 3 residue, a modification carried out by ghrelin O-acyl transferase (GOAT)^29^. Although des-acyl ghrelin (DAG) is considered a degradation product of ghrelin with no biological activity, DAG may antagonize or support ghrelin’s activities, or function completely independently^30,31^. In PD, while acylated ghrelin has neuroprotective effects, the role of des-acyl ghrelin has not yet been well clarified^32^. Nonetheless, the literature is heterogeneous, with some but not all studies not having observed significant differences in fasting concentrations of total^33^ and active ghrelin^34^, or in the postprandial concentrations of both^35^.

Considering the conflicting results, a meta-analysis of the available literature is essential to clarify the magnitude of ghrelin dysregulation in PD. To date, no meta-analysis has addressed this aspect. In addition to quantifying the effect of PD on ghrelin, we intend to explore factors contributing to potential variations in ghrelin concentrations and identify sources of heterogeneity across studies. A better understanding may help to refine the role of ghrelin as an important biomarker and its implications for pathophysiology, nutritional management, and pharmacological therapy in PD.

## Results

### Identified articles

**Fig. 1:**
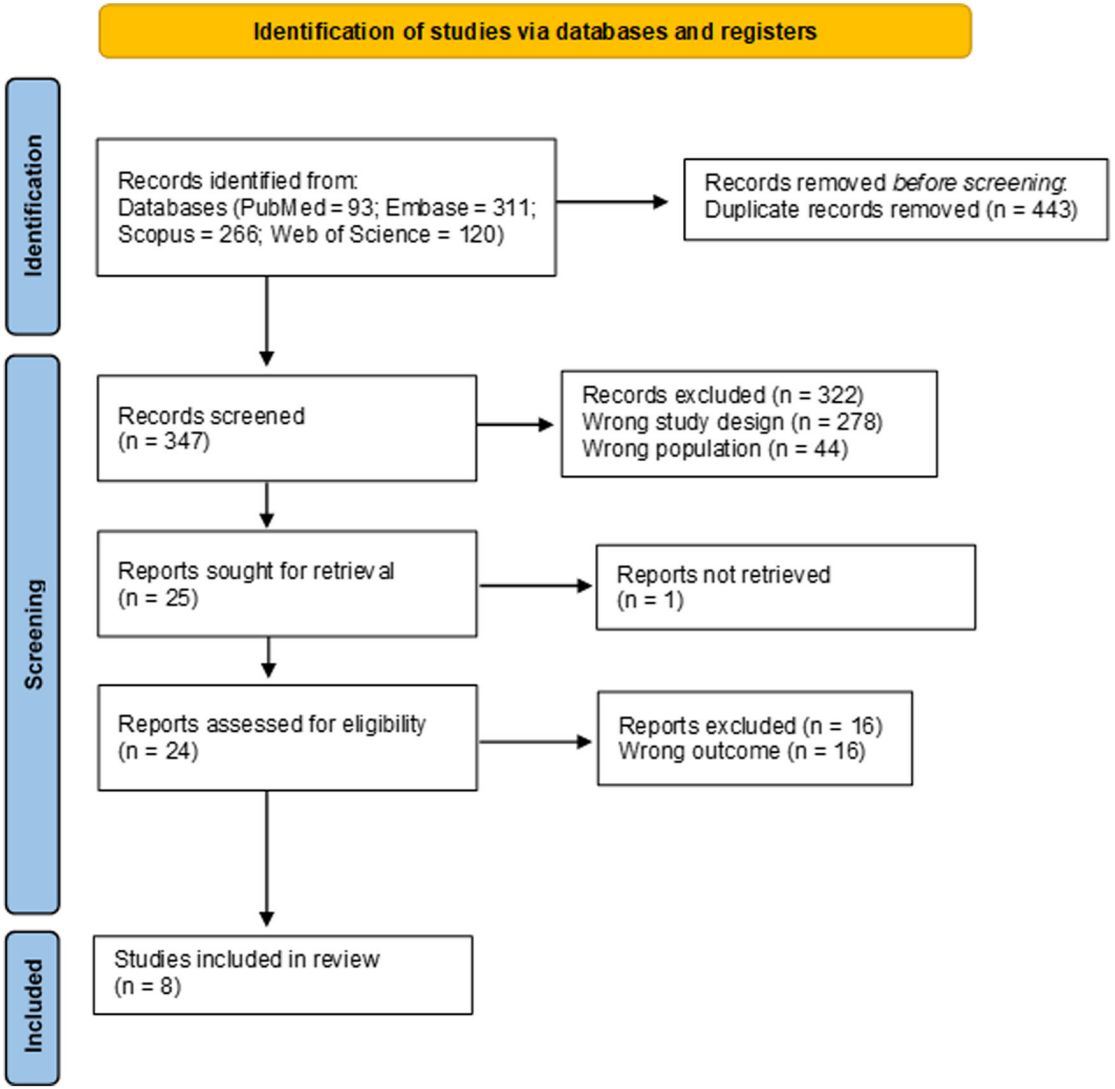
PRISMA flow chart for study selection. This flow diagram shows the study selection process. A total of 790 articles were identified. After removing 443 duplicates, the titles and abstracts of 347 articles were screened. A total of 24 articles were considered for inclusion, and eight of these were selected.

After searching the selected databases, we identified 790 articles. Initially, we excluded 443 articles because they were duplicates, identified through the Rayyan platform (https://new.rayyan.ai/), which enables the manual evaluation of overlapping records from different databases. We read the titles and abstracts of 347 articles. After this, 24 articles remained for a full assessment of eligibility. We excluded 16 studies after full reading and included 8 in the final analysis. All the included articles were written in English. Figure 1 shows the flowchart that presents in detail the selection steps of the articles.

### Risk of bias

We evaluated the risk of bias in the quasi-experimental studies using the JBI (Table S1)^26,33,35–38^. Considering that all the studies included individuals already diagnosed with PD, we classified the risk of bias related to temporal precedence as “unclear”, i.e., there is no confusion about which variable comes first. However, considering that the objective of the studies was not to assess causality, we judge this characteristic not to impair the internal validity of the studies. We evaluated bias related to selection and allocation based on the presence of a control group. In this domain, all the studies showed a low risk of bias and good internal validity by comparing the results of the PD group with controls. In the assessment of bias related to confounding factors, most studies showed a low risk of bias and good internal validity when comparing similar groups in aspects such as age, sex, weight, and body mass index^26,33,35–37^, with only one study showing significant differences between the groups^38^. Regarding bias related to the administration of intervention/exposure, we considered studies that withheld any type of intervention at the time of blood collection to be at low risk of bias and greater internal validity. We classified two studies as low risk of bias^35,37^. Three studies showed lower internal validity by not controlling for this factor^26,33,36^, while in one study this information is unclear^38^. We assessed bias related to assessment, detection, and measurement of the outcome on the following basis: whether multiple measurements of the outcome were taken after the “intervention” (meal); whether the comparisons were measured in the same way; and whether the outcomes were measured reliably. Firstly, only one study did not evaluate postprandial ghrelin concentrations at different time points^38^, while the other studies carried out at least four evaluations^26,33,35–37^, which were considered to have a low risk of bias and greater internal validity. In the second case, all outcomes were measured in the same way in the compared groups, meaning that all studies presented a low risk of bias and good internal validity. On the last point, we consider that all the studies assessed the outcomes reliably and were classified as low risk of bias and good internal validity^26,33,35–38^. We assessed the risk of bias related to participant retention based on incomplete follow-up and classified all the studies as having a low risk of bias and good internal validity. Finally, we assessed the validity of the statistical conclusion, and most of the studies presented a low risk of bias^33,35–37^, with two studies presenting limited information^26,38^. Thus, we judged only one study as having a moderate to high risk of bias and lower internal validity^38^. We considered all the other studies to have a low risk of bias and good internal validity^26,33,35–37^.

We assessed the cross-sectional studies using the tool proposed by the SURE (Table S2). The two studies evaluated were rated similarly and presented an overall low risk of bias due to compliance with most of the domains assessed, including: definition of the question and objectives; eligibility, selection and characteristics of the participants; measures and description of the results; conflicts of interest; and limitations. Conversely, they presented limited information regarding the description of the statistics and lacked details about the study size^34,39^. One of the studies also provided limited information on setting, locations and relevant dates^34^.

### Effects of PD on ghrelin

Data on methodological and clinical characteristics are shown in Table 1. We classified six studies as quasi-experimental due to the experimental design including longitudinal evaluations after an intervention^26,33,35–38^ while two studies were cross-sectional because the evaluation was carried out at just one point in time^34,39^. Considering the groups included according to the inclusion criteria, a total of 985 participants were included in the studies, with a minimum of 28^34^ and a maximum of 594^26^ participants, all of whom were adults. Six studies evaluated male and female individuals^26,33,34,36,37,39^, while two did not report sex^35,38^. The time of diagnosis of the participants’ PD and the treatment used at the time of participation in the study were indicated in five^34,36–39^ and three^33,34,36^ studies, respectively. Two studies indicated that therapy was interrupted for the evaluations^35,37^ while three studies did not report the therapy used at the time of the evaluations or whether it was interrupted^26,38,39^. Only one study included groups of participants with PD who were treated and drug-naïve^33^.

**Table 1:** Characteristics of the included studies

Authors	Study design	Population	Sex	Age
Fiszer et al.^34^	Cross-sectional	Control (n = 12) PD (n = 16) Duration of PD (6.62 ± 3.79 years) Therapy = Levodopa (715.62 ± 648.52 mg/day)	Control (F = 9; M = 3) PD (F = 8; M = 8)	Control = 58.58 ± 8.60 years PD = 60.5 ± 8.65 years
Unger et al.^33^	Quasi-experimental	Control (n = 20) PD (n = 19 drug-naive; n = 20 under therapy) Duration of PD (Drug-naive = median 1.1 [95% CI: 0.6–3.1]; Under therapy = median 3.9 [95% CI: 1.9–7.3]) Therapy = levodopa monotherapy (n = 2), levodopa plus entacapone (n = 1), levodopa plus selegiline (n = 1), levodopa plus pramipexole (n = 2), levodopa plus entacapone plus pramipexole plus amantadine (n = 1), levodopa plus rotigotine (n = 2), cabergoline plus rasagiline (n = 1), pramipexole monotherapy (n = 4), pramipexole plus selegiline plus budipine (n = 1), pramipexole plus rotigotine (n = 1), ropinirol monotherapy (n = 1), and rotigotine monotherapy (n = 2)	Control (F = 8; M = 12) Drug-naive PD (F = 7; M = 12) Under therapy PD (F = 11; M = 9)	Control = 55.2 ± 8.8 years Drug-naive PD = 62.4 ± 11.2 years Under therapy PD = 63.7 ± 8.5 years
Song et al.^26^	Quasi-experimental	Control (n = 303: 20 for postprandial) PD (n = 291; 20 for postprandial) Duration of PD = Not informed (PD stages 1–3) Therapy = Not informed	Control (F = 144; M = 159) PD (F = 131; M = 160)	Control = 66.04 ± 9.89 years PD = 65.96 ± 9.95 years
Tarianyk et al.^38^	Quasi-experimental	Control (n = 21) Akinetic-rigid PD (n = 16) Mixed PD (n = 14) Duration of PD (Akinetic-rigid = 6.42 ± 2.13 years; Mixed = 9.13 ± 1.87 years) Therapy = Not informed	Not informed	Control = 58.63 ± 6.16 years Akinetic-rigid PD = 59.94 ± 7.97 years Mixed PD = 62.13 ± 10.39 years
Pietraszko et al.^37^	Quasi-experimental	Control (n = 30) PD (n = 29) Duration of PD (10.9 ± 4.7 years) Therapy = Not informed (All medications were withheld or refused by the patient, ensuring a drug-free period of at least 12 h)	Control (F = 11; M = 19) PD (F = 11; M = 18)	Control = 58.4 ± 11.1 years PD = 59.4 ± 9.9 years
Hornsby et al.^35^	Quasi-experimental	Control (n = 20) PD (n = 20) PD Dementia (n = 8) Duration of PD (Not informed) Therapy = Not informed (All medications were withheld for plasma collection)	Control (F = 9; M = 11) PD (F = 9; M = 11) PD Dementia (F = 1; M = 7)	Control = 74.0 ± 6.28 years PD = 72.2 ± 5.51 years PD Dementia = 74.75 ± 5.99 years
Siervo et al.^36^	Quasi-experimental	Control (n = 20) PD (n = 19) PD-CI (n = 16) Duration of PD (PD = 69.5 ± 69.0 months; PD-CI = 107.3 ± 59.5 months) Therapy = Standard preparation levodopa (n = 16 PD; n = 16 PD-CI), Controlled release levodopa (n = 3 PD; n = 9 PC-CI), Dopamine agonists (n = 7 PD), MAOIB (n = 11 PD; n = 2 PD-CI), COMT inhibitors (n = 4 PD; n = 7 PD-CI), Cholinesterase inhibitors (n = 7 PD-CI), Selective anticholinergics (n = 3 PD; n = 3 PD-CI), Antidepressants (n = 7 PD-CI), Pro-appetitive medicines (n = 3 PD-CI), and Anorectic medications (n = 11 PD; n = 6 PD-CI). All medications were withheld for plasma collection.	Control (F = 9; M = 11) PD (F = 9; M = 10) PD-CI (F = 7; M = 9)	Control = 74.0 ± 6.2 years PD = 72.5 ± 5.5 years PD-CI = 74.3 ± 6.0 years
Majeed; Al-Lami; AlGawwam^39^	Cross-sectional	Control (n = 40) PD (n = 51) Duration of PD (<5 years = 36 [70.6%]; 5–10 years = 12 [23.5%]; >10 years = 3 [5.9%]) Therapy = Not informed	Control (F = 25; M = 15) PD (F = 13; M = 38)	Control = 41.25 ± 18.30 PD = 63.76 ± 12.29

*PD* Parkinson Disease, *PD-CI* PD with cognitive impairment, *F* Female, *M* Male, *MAOIB* Monoamine oxidase inhibitors-B, *COMT* catechol-O-methyltransferase inhibitor.

A total of three studies assessed fasting and postprandial concentrations of total and active ghrelin^26,35,36^ (Table 2). First, fasting patients with PD of both sexes, exhibited significantly reduced total and active ghrelin concentrations when compared to the respective controls. There were no differences between the different levels of PD (1, 1.5, 2, 2.5, and 3). In healthy controls, females had higher fasting total and active ghrelin levels than males, while in PD, females had higher fasting total ghrelin levels than males. Also, both postprandial total and active ghrelin levels were attenuated in PD^26^. In another study, PD or PD associated with dementia (PDD) groups did not exhibit significant differences in fasting concentrations of total and active ghrelin when compared with controls. The PD group also showed no changes in postprandial (180 min) ghrelin concentrations when compared to controls. However, the acylated/unacylated ghrelin ratio was reduced under postprandial conditions in the PDD group when compared to the control and PD groups^35^. In the last study to evaluate both total and active ghrelin, groups with PD or PD with cognitive impairment (PD-CI) did not show any significant difference in fasting or postprandial levels compared to controls^36^.

**Table 2:** Plasma fasting ghrelin concentrations

Authors	Fasting total ghrelin	Fasting active ghrelin
Fiszer et al.^34^	NA	**Mean** **±** **SD (pg/ml)** Control = 154.45 ± 109.13 vs PD = 117.63 ± 67.68; *p*: n.s.
Unger et al.^33^	**Mean** **±** **SD (pg/ml)** Control = 970 ± 324 vs Drug-naive PD = 857 ± 249; *p*: n.s. Control *vs* Under therapy PD = 849 ± 221; *p*: n.s.	NA
Song et al.^26^	**Mean** **±** **SEM (pg/ml)**^a^ **Male** Control = 514.16 ± 16.91 vs PD = 453.28 ± 11.84; *p* < 0.05 **Female** Control = 649.47 ± 25.37 vs PD = 539.53 ± 18.60; *p* < *0.05*	**Mean** **±** **SEM (pg/ml)**^a^ **Male** Control = 122.12 ± 9.29 vs PD = 81.85 ± 5.76; *p* < *0.05* **Female** Control = 149.11 ± 10.18 vs PD = 96.01 ± 7.08; *p* < *0.05*
Tarianyk et al.^38^	**Median (Q1-Q3) (pg/ml)**^a^ Control = 1521.21 (1305.2–1666.67) vs Mixed PD = 1275.33 (1132.68–1500.87); *p* = 0.029 Control vs Akinetic-rigid PD = 1245.02 (864.94–1379.66); *p* = 0.044	NA
Pietraszko et al.^37^	NA	**Mean** **±** **SD (pg/ml)** Control = 260.29 ± 87.82 *vs* PD = 177.53 ± 68.01; *p* = 0.0003
Hornsby et al.^35^	**Mean** **±** **SEM (pg/ml)** Control = 541.0 ± 87.98 vs PD = 647.9 ± 108.7; *p* = n.s. Control *vs* PD Dementia = 377.8 ± 77.00; *p* = n.s.	**Mean** **±** **SEM (pg/ml)** Control = 78.93 ± 13.77 vs PD = 114.4 ± 18.82; *p* = n.s. Control vs PD Dementia = 51.39 ± 17.24; *p* = n.s.
Siervo et al.^36^	**EMM** ± **SE (pg/ml)**^a^ Control = 6.27 ± 0.49 vs PD = 6.04 ± 0.34; *p*: n. s. Control vs PD-CI = 5.96 ± 0.43; *p*: n. s.	**EMM** ± **SE (pg/ml)**^a^ Control = 4.31 ± 0.43 vs PD = 4.14 ± 0.28; *p*: n. s. Control vs PD-CI = 4.35 ± 0.35; *p*: n. s.
Majeed; Al-Lami; AlGawwam^39^	**Plasma total ghrelin serum concentrations (ng/ml)** Mean ± SD Control = 8.7 ± 2.1 vs PD = 2.8 ± 1.5; *p* < 0.001	NA

*PD* Parkinson Disease, *PD-CI* PD with cognitive impairment, *SD* Standard deviation, *SEM* Standard error of the mean, 95% *CI* Confidence interval, *Q1* First quartile is the 25th percentile of the data set, *Q3* Third quartile is the 75th percentile of the data set, *EMM* Estimated marginal means, *SE* Standard error, *NA* Not assessed.

^a^The data was transformed by estimation into mean ± SD for the meta-analysis.

Three studies assessed only total ghrelin^33,38,39^, two of which also evaluated postprandial concentrations^33,38^. First, drug-naïve PD and PD under therapy exhibited a less pronounced recuperation of postprandial ghrelin levels compared to controls, but there were no significant differences in fasting ghrelin serum concentrations between the groups^33^. Furthermore, when the treated and drug-naïve PD groups were evaluated separately, significant differences compared to the control group were observed only in the treated ones^33^. Next, both individuals with mixed PD or akinetic-rigid PD exhibited significantly lower fasting and postprandial concentrations of total ghrelin compared to control. Interestingly, there was a reduction in postprandial ghrelin concentrations only in the akinetic-rigid PD group when compared to the control, as well as reductions in postprandial (evening) concentrations when compared to fasting (morning) concentrations in the akinetic-rigid PD group and as well as in the control group^38^. Finally, individuals with PD showed significantly lower fasting concentrations of total ghrelin compared to controls^39^.

Lastly, two studies evaluated only active ghrelin^34,37^, and one of them also evaluated postprandial^37^. In the first, individuals with PD did not show significant reductions in fasting concentrations of active ghrelin^34^, while the second found significant reductions in fasting and postprandial concentrations of active ghrelin in individuals with PD^37^, always compared to controls.

**Fig. 2:**
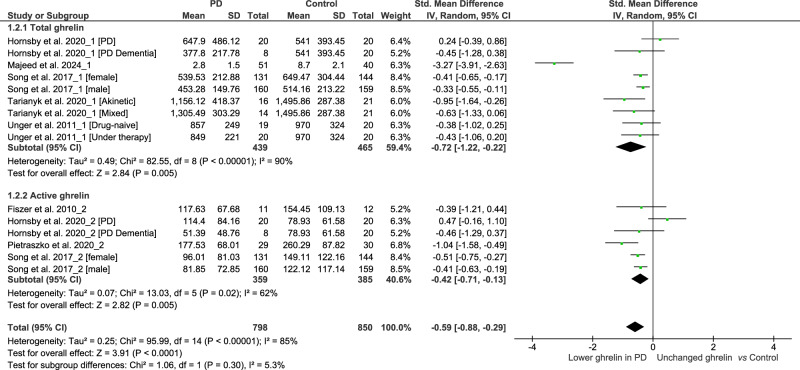
Forest plot of the included studies evaluating fasting ghrelin levels. Plasma levels of active and total ghrelin in fasting patients with PD and in the control group were compared using standardized mean differences (SMD) and 95% confidence intervals. Significant reductions in both total (SMD: −0.72, 95% CI: −1.22, −0.22; Z: 2.84; P: 0.005) and active (SMD: −0.42, 95% CI: −0.71, −0.13; Z: 2.82; P: 0.005) ghrelin were observed in patients with PD. High heterogeneity was found for total ghrelin (Tau² = 0.49, Chi² = 82.55, df = 8, P < 0.00001, I² = 90%), while moderate heterogeneity was found for active ghrelin (Tau² = 0.07, Chi² = 13.03, df = 5, P = 0.02, I² = 62%). No significant subgroup differences were found (Chi² = 1.06, df = 1, P = 0.30; I² = 5.3%).

**Fig. 3:**
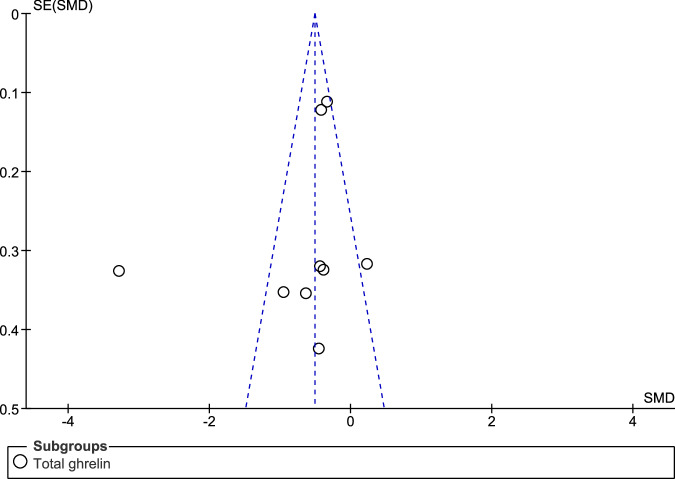
Funnel plot of included studies for fasting total ghrelin. The slightly asymmetrical funnel plot indicates that there is possible publication bias for fasting total ghrelin.

**Fig. 4:**
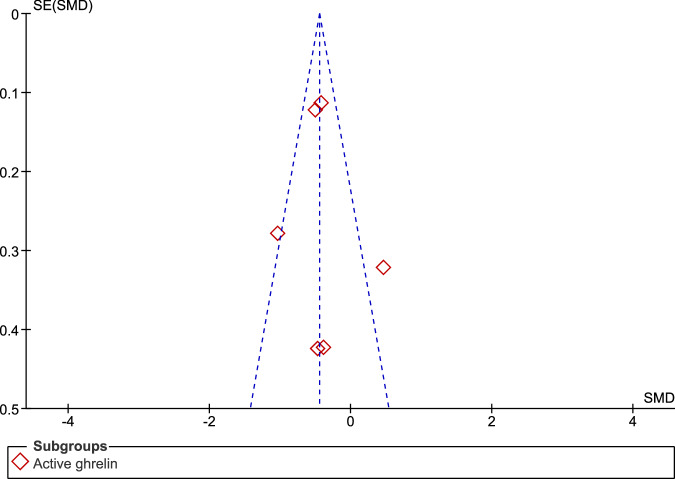
Funnel plot of included studies for fasting active ghrelin. The symmetrical funnel plot indicates that there is no apparent publication bias for fasting active ghrelin.

When compared to controls, fasting individuals with PD exhibited a significant reduction in the concentrations of total ghrelin (SMD: −0.72, 95% CI −1.22, −0.22; Z = 2.84; P = 0.005) and active ghrelin (SMD: −0.42, 95% CI −0.71, −0.13; Z = 2.82; P = 0.005). We observed high heterogeneity in the assessment of total ghrelin (Tau² = 0.49; Chi² = 82.55, df = 8, p < 0.00001; I² = 90%), and moderate heterogeneity in the assessment of active ghrelin (Tau² = 0.07; Chi² = 13.03, df = 5, P = 0.02; I² = 62%). There was no significant difference between the two subgroups (Chi² = 1.06, df = 1, P = 0.30; I² = 5.3%) (Fig. 2). The funnel plot showed some asymmetry in the evaluation of total ghrelin (Fig. 3), indicating a possible publication bias, but not in the evaluation of active ghrelin (Fig. 4).

**Fig. 5:**
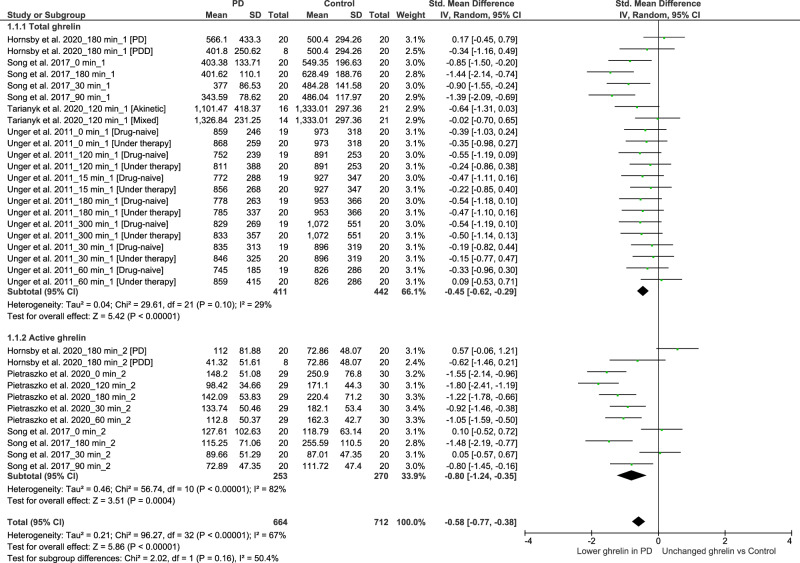
Forest plot of included studies evaluating postprandial ghrelin. Postprandial plasma levels of active and total ghrelin in patients with PD and in the control group were compared using standardized mean differences (SMD) and 95% confidence intervals. Significant reductions were observed in both total (SMD: −0.45, 95% CI −0.62, −0.29; Z = 5.42; P < 0.00001) and active (SMD: −0.80, 95% CI −1.24, −0.35; Z = 3.51; P = 0.0004) ghrelin. Moderate heterogeneity was found for total ghrelin (Tau² = 0.04; Chi² = 29.61, df = 21, P = 0.10; I² = 29%), while high heterogeneity was observed for active ghrelin (Tau² = 0.46; Chi² = 56.74, df = 10, P < 0.00001; I² = 82%). There were no significant subgroup differences (Chi² = 2.02, df = 1, P = 0.16; I² = 50.4%).

**Fig. 6:**
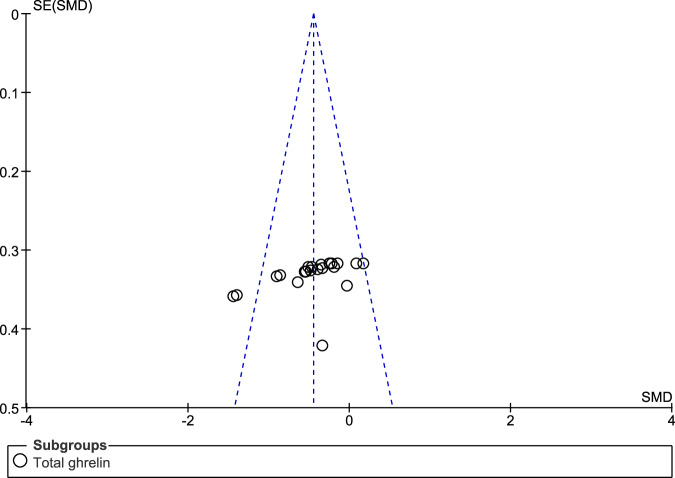
Funnel plot of included studies for postprandial total ghrelin. The symmetrical funnel plot indicates that there is no apparent publication bias for postprandial total ghrelin.

**Fig. 7:**
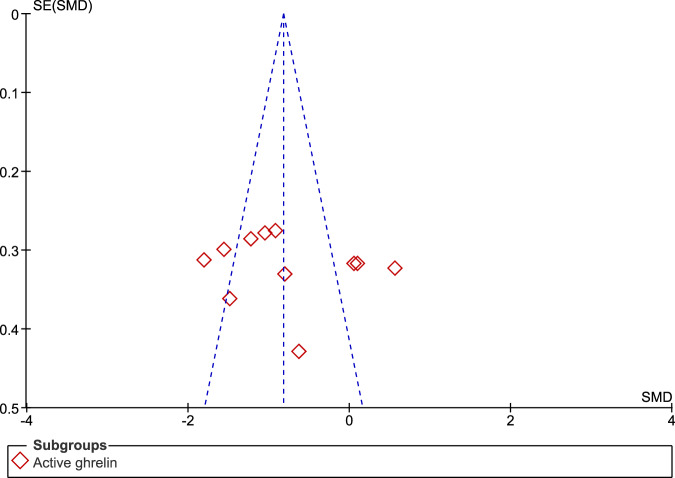
Funnel plot of included studies for postprandial active ghrelin. The symmetrical funnel plot indicates that there is no apparent publication bias for postprandial active ghrelin.

When compared to controls, individuals with PD exhibited significantly reduced concentrations of total ghrelin (SMD: −0.45, 95% CI −0.62, −0.29; Z = 5.42; P < 0.00001) and active ghrelin (SMD: −0.80, 95% CI −1.24, −0.35; Z = 3.51; P = 0.0004) postprandially. We observed moderate heterogeneity in the assessment of total ghrelin (Tau² = 0.04; Chi² = 29.61, df = 21, P = 0.10; I² = 29%), and high heterogeneity in the assessment of active ghrelin (Tau² = 0.46; Chi² = 56.74, df = 10, P < 0.00001; I² = 82%). There was no significant difference between the two subgroups (Chi² = 2.02, df = 1, P = 0.16; I² = 50.4%) (Fig. 5). The funnel plot showed good asymmetry in both evaluations (Figs. 6 and 7).

## Discussion

Here we report the results of a meta-analysis on changes in ghrelin concentrations in individuals with PD. We found that individuals with PD exhibited a significant reduction in both fasting and postprandial concentrations of total and active ghrelin, with similar effect sizes.

Although we observed a robust reduction of ghrelin concentrations in PD, not all studies reported significant reductions, which may suggest important methodological differences. A total of six studies evaluated fasting total ghrelin concentrations, with three indicating significant reductions. Two of the studies that observed significant reductions had the largest total number of subjects^26,39^. Five studies evaluated fasting active ghrelin concentrations and only two reported significant reductions, both with the largest number of subjects per group^26,37^. Thus, the relatively small sample size of some studies may have been responsible for the lack of statistical significance, as suggested in some of the included studies^26,33^.

A further important aspect may be related to the use of medication. Some of the studies indicated that the medication was discontinued at the time of blood collection, while others indicated that the individuals were under therapy or did not provide information on this aspect. Only one of the included studies divided groups of treated and drug-naïve individuals^33^. Interestingly, when comparing both groups with controls, only the treated group showed significant changes^33^. The differences may have been caused by the sample size and statistical corrections, rather than the use of antiparkinsonian drugs. Nevertheless, many therapies used for PD influence concentrations of dopamine, which is closely related to ghrelin signaling^40^; high doses dopamine influence ghrelin secretion in vitro^41^. Future studies should investigate this topic by including both treated and untreated patients, to assess the influence of antiparkinsonian drugs on ghrelin concentrations. In studies where all the subjects have already started drug treatment due to the duration of PD, blood samples can be collected by stratifying the groups based on whether therapy has been suspended for a period of at least 12 h. A crucial area for the interaction between dopamine and ghrelin may be the VTA. This area contains a subpopulation of dopaminergic neurons that project to areas such as the amygdala, the prefrontal cortex, the hippocampus, and the nucleus accumbens^42,43^. The VTA responds to food related odors^44^; ghrelin influences the VTA’s reactivity to food, playing an important role in the hedonic control of eating^16^. Injecting ghrelin directly into the VTA of Sprague-Dawley (SD) rats and C57BL/6 mice led to changed behavior, including an increased level of work to obtain food rewards^42,45^. In healthy humans, intravenous administration of ghrelin increased reward-related activity in dopamine-responsive areas during odor conditioning, and rendered cues associated with food odor more pleasant^16^.

In this context, it is essential to highlight the role of ghrelin on olfaction in the context of eating behavior. In fact, there is a bidirectional relationship between ghrelin and olfaction. While ghrelin administration increases the pleasantness of food odors^16^, olfactory stimulation during the cephalic phase of feeding stimulates ghrelin secretion^46^. Ghrelin receptors are found in structures of the olfactory bulb, such as glomeruli, mitral cells and granule cells, providing the transmission of signals to the hypothalamus and amygdala^17,18^. This axis seems to be affected in PD. As previously mentioned, approximately 90% of individuals with PD have altered olfactory function^9^. Olfactory dysfunction occurs early, up to 10 to 20 years before the onset of motor disorders and the diagnosis of PD^10^. Olfactory function is also directly related to the availability of dopamine transporters in the hippocampus, amygdala, and striatum^47^. Future studies could investigate olfactory function with appropriate behavioral tools, such as the Sniffin’Sticks while assessing serum ghrelin concentrations in PD^48^.

The interplay between ghrelin and dopamine extends to the gastrointestinal system, as ghrelin is essential for maintaining gastrointestinal functions, including regulation of motility, protection of mucosal tissue, regulation of glucose, and lipid metabolism^14^. Gastrointestinal dysfunctions, including IBS, dysphagia, gastroparesis and constipation are common non-motor symptoms in individuals with PD^49–52^. In turn, dopamine stimulates exocrine secretions and modulates mucosal blood flow^53,54^. PD patients with chronic constipation exhibit reduced myenteric dopaminergic neurons in the colon^55^, as well as alpha-synuclein inclusions in enteric dopaminergic neurons^56^, suggesting a key role in gastrointestinal pathogenesis. Therefore, understanding the different relationships between ghrelin and dopamine may be useful for developing more effective interventions, including drug therapy involving ghrelin itself, but also dietary interventions. In SD rats with PD induced by 6-hydroxydopamine (6-OHDA), treatment with acyl‑ghrelin 7 days before injury protected dopaminergic neurons in the SNpc region of the midbrain^57^. Treatment with acyl-ghrelin, but not des-acyl ghrelin, attenuated the MPTP-induced loss of tyrosine hydroxylase neurons in the nigrostriatal pathway and reduced microglial activation in the substantia nigra in ghrelin KO C57/Bl6 mice^32^. Treatment with a ghrelin agonist (HM01) prevented levodopa-induced gastric transit delay in 6-OHDA-induced PD in SD rats, besides relieving constipation^58^. Regarding nutrition, while it is still too early to define the best intervention, it is known that dietary factors can influence the risk of developing PD, reduce symptoms, and potentially modify the progression of the disease in diagnosed individuals^59^. Nevertheless, studies on humans are needed to evaluate the efficacy of ghrelin treatment and different dietary interventions on both the development and progression of PD.

As mentioned above, ghrelin plays an important role in glucose regulation, which may be linked to its function in improving insulin signaling. In an in vitro study, neurons treated with ghrelin showed increased glucose uptake and improved tau hyperphosphorylation^60^. Mechanistically, ghrelin activated the phosphorylation of key proteins such as Akt and GSK-3β^60^. In this context, there is evidence that individuals with PD also have central insulin resistance (IR)^61^. An in vitro study using a midbrain organoid model demonstrated that prolonged exposure to high concentrations of insulin caused a reduction in dopaminergic neurons and metabolic alterations^62^. Furthermore, diabetes-associated IR promoted the development and progression of PD through mitochondrial dysfunction and increased production of reactive oxygen species. Therefore, while our review highlights ghrelin as a crucial hormone in PD, a broader evaluation of different hormones potentially implicated in PD is needed.

Almost all the studies included both sexes. However, only one study evaluated fasting concentrations of total and active ghrelin separately between them. In this study, fasting total plasma ghrelin levels were higher in healthy women than in men. Patients with PD showed the same trend, but there was no significant difference^26^. Other studies have also observed significant differences between the sexes, including age as a significant indicator after adjusting for sex, fat mass and body size in healthy individuals^63^. Sex is one of the most important risk factors for PD as the risk of developing PD can be up to twice as high in men; however, women have a higher mortality rate and faster disease progression^64–66^. Different responses and symptoms associated with drug therapy or deep brain stimulation are also observed between the sexes^67^. Therefore, the possible sex differences in ghrelin concentrations should be investigated in future studies.

According to Braak, the progression of PD is divided into six neuropathological stages, with the continuous development of Lewy neurites and bodies^68^. The first phase involves the dorsal motor nucleus of the vagal nerve^68^. Although ghrelin is a hormone primarily derived from the stomach, studies have shown that the integrity of the vagus nerve is necessary for its functions^69^, but there is still debate about this in humans^70^. In PD, bilateral atrophy of the vagus nerve was observed in patients with a PD duration of 10.1 ± 7.4 years^71^. Accumulation of α-synuclein in the vagus nerve was observed after autopsy in 89% of PD patients (average PD duration: 13 years)^72^. In this context, only one study evaluated possible differences in fasting total and active ghrelin levels between PD stages (1, 1.5, 2, 2.5, and 3), with no significant difference found^26^. Future studies should consider assessing ghrelin levels at different stages, including preclinical stages of PD.

This review has some limitations. The low number of studies included, and the high heterogeneity indicated by the meta-analysis suggest that the results should be interpreted cautiously. In the meta-analysis, data from one study were not included due to inadequate format, and data from another study were incompletely included regarding postprandial ghrelin. However, the magnitude of the effect was high in all the analyses, reinforcing the relevance of the results.

The results presented corroborate ghrelin as a relevant hormone in PD, with significant reductions in plasma concentrations of total and active ghrelin in fasting or postprandial states, representing the first systematic review and meta-analysis on the subject. By exploring the different characteristics of the studies, the results also highlights the importance of investigating the effects of sex, pharmacological treatment, and disease progression on ghrelin levels, as well as exploring its therapeutic potential. In addition, broader hormonal assessments – beyond ghrelin – are needed to better understand the complex neuroendocrine mechanisms involved in the pathophysiology of PD. A deeper understanding could contribute to diagnosis and the development of therapies.

## Methods

### Literature search

The literature search was carried out in February 2025 in the following databases: Medline/PubMed (1966–2025), SCOPUS (1969–2025), EMBASE (1947–2025), and Web of Science (1900–2025). The search was carried out individually by two authors (Gouveia, H. J. C. B. and Santos-Junior, O. H.). A third reviewer was consulted in case of disagreements (Frasnelli, J.). The search was carried out in the databases using the following descriptors: “Parkinson Disease [MeSH]” OR “Parkinsonian Disorders [MeSH]” OR “Parkinson’s Disease [non-MeSH]” AND “Ghrelin [MeSH]”. The protocol for this review is published in the international prospective register of systematic reviews (PROSPERO) database (ID: CRD420250655742).

### Study selection and data extraction

We selected the studies in two stages (Gouveia, H. J. C. B. and Santos-Junior, O. H.). In the first stage, the selection was made by reading the titles and abstracts of the original studies selected for the research. We excluded studies based on the following criteria: (a) wrong study design; and (b) wrong population. In the second phase, we read the selected articles in full. Then we adopted the following exclusion criteria: (a) wrong outcome. We included quasi-experimental and observational studies assessing fasting and postprandial plasma concentrations of total and/or active ghrelin in individuals with PD and controls. We excluded studies and/or groups in which participants received interventions other than pharmacological therapy for the treatment of PD. In addition, we did not include studies in which participants had other chronic conditions. For the search and selection of articles, there were no restrictions on the year of publication or the language of the article.

Data extraction was performed by two reviewers (Gouveia, H. J. C. B. and Santos-Junior, O. H.). We extracted the following general data on the studies: Authors and year of publication. Population characteristics included: The number of participants per group, the duration and therapy of PD, and the gender and age of the participants (Table 1). Regarding the outcome, we collected data on: Fasting plasma concentrations of total and active ghrelin (pg/ml or ng/ml), and postprandial plasma concentrations of total and active ghrelin (pg/ml, 0–300 min). We present the *p*-values in the table when comparisons were made between the PD and control groups (Tables 2 and 3) and contacted the authors by e-mail in the event of missing or incomplete data.

**Table 3:** Plasma postprandial ghrelin concentrations

Authors	Postprandial total ghrelin	Postprandial active ghrelin
Fiszer et al.^34^	NA	NA
Unger et al.^33^	**Mean** **±** **SD (pg/ml)** **0** **min;** ***p*** = ***n.s****.* Control = 973 ± 318 vs Drug-naive PD = 859 ± 246 Control vs Under therapy PD = 868 ± 259 **15** **min;** ***p*** = **n.s**. Control = 927 ± 347 vs Drug-naive PD = 772 ± 288 Control vs Under therapy PD = 856 ± 268 **30** **min;** ***p*** = **n.s**. Control = 896 ± 319 vs Drug-naive PD = 835 ± 313 Control vs Under therapy PD = 846 ± 325 **60** **min;** ***p*** = **n.s**. Control = 826 ± 286 vs Drug-naive PD = 745 ± 185 Control vs Under therapy PD = 859 ± 415 **120** **min;** ***p*** = **n.s**. Control = 891 ± 253 vs Drug-naive PD = 752 ± 239 Control vs Under therapy PD = 811 ± 388 **180** **min;** ***p*** = **0.033** Control = 953 ± 336 vs Drug-naive PD = 778 ± 263 Control vs Under therapy PD = 785 ± 337 **300** **min;** ***p*** = **n.s**. Control = 1072 ± 551 vs Drug-naive PD = 829 ± 269 Control vs Under therapy PD = 833 ± 357	NA
Song et al.^26^	**Mean** **±** **SEM (pg/ml)**^a^ **0** **min** Control = 549.35 ± 43.97 vs PD = 403.38 ± 29.90 **30** **min** Control = 484.28 ± 31.66 *vs* PD = 377.00 ± 19.35 **90** **min** Control = 486.04 ± 26.38 *vs* PD = 343.59 ± 17.58 **180** **min** Control = 628.49 ± 42.21 *vs* PD = 401.62 ± 24.62	**Mean** **±** **SEM (pg/ml)**^a^ **0** **min** Control = 118.79 ± 14.12 vs PD = 127.61 ± 22.95 **30** **min** Control = 87.01 ± 10.59 vs PD = 89.66 ± 11.47 **90** **min** Control = 111.72 ± 10.60 vs PD = 72.89 ± 10.59 **180** **min** Control = 255.59 ± 24.71 vs PD = 115.25 ± 15.89
Tarianyk et al.^38^	**Median (Q1-Q3) (pg/ml)**^a^ 120 min Control = 1365.37 (1133.33–1507.36) *vs* Mixed PD = 1382.69 (1165.59–1446.33); *p* = n.s. Control *vs* Akinetic-rigid PD = 987.01 (896.97–1393.08); *p* = 0.047	NA
Pietraszko et al.^37^	NA	**Mean** **±** **SD (pg/ml)** **0** **min;** ***p*** = **0.0001** Control = 250.9 ± 76.8 vs PD = 148.2 ± 51.08 **30** **min;** ***p*** = **n.s**. Control = 182.1 ± 53.4 vs PD = 133.74 ± 50.46 **60** **min;** ***p*** = **0.042** Control = 162.3 ± 42.7 vs PD = 112.80 ± 50.37 **120** **min;** ***p*** = **0.002** Control = 171.1 ± 44.3 vs PD = 98.42 ± 34.66 **180** **min;** ***p*** = **0.0001** Control = 220.4 ± 71.2 vs PD = 142.09 ± 53.83
Hornsby et al.^35^	**Mean** **±** **SEM (pg/ml)**^a^ **180** **min;** ***p*** = **n.s**. Control = 500.4 ± 294.26 vs PD = 566.1 ± 433.3 Control vs PD Dementia = 401.8 ± 250.62	**Mean** **±** **SEM (pg/ml)**^a^ **180** **min;** ***p*** = **0.0169** Control = 72.86 ± 48.07 vs PD = 112.0 ± 81.88 Control vs PD Dementia = 41.32 ± 51.61
Siervo et al.^36^	**EMM** ± **SE (pg/ml)** **5** **min;** ***p*** = **n.s**. Control = 6.23 ± 0.43 *vs* PD = 5.98 ± 0.30 Control vs PD-CI = 6.67 ± 0.77 **15** **min;** ***p*** = **n.s**. Control = 6.19 ± 0.48 vs PD = 5.92 ± 0.35 Control vs PD-CI = 5.86 ± 0.40 **30** **min;** ***p*** = **n.s**. Control = 6.16 ± 0.37 vs PD = 5.87 ± 0.25 Control vs PD-CI = 5.96 ± 0.32 **60** **min;** ***p*** = **n.s**. Control = 5.95 ± 0.36 vs PD = 5.81 ± 0.25 Control vs PD-CI = 5.88 ± 0.31 **120** **min;** ***p*** = **n.s**. Control = 6.02 ± 0.38 vs PD = 5.70 ± 0.26 Control vs PD-CI = 5.92 ± 0.33 **180** **min;** ***p*** = **n.s**. Control = 6.22 ± 0.40 vs PD = 5.81 ± 0.28 Control vs PD-CI = 6.12 ± 0.35	**EMM** ± **SE (pg/ml)** **5** **min;** ***p*** = **n.s**. Control = 4.19 ± 0.44 vs PD = 3.99 ± 0.29 Control vs PD-CI = 4.02 ± 0.36 **15** **min;** ***p*** = **n.s**. Control = 4.19 ± 0.45 vs PD = 3.83 ± 0.29 Control vs PD-CI = 3.57 ± 0.35 **30** **min;** ***p*** = **n.s**. Control = 3.55 ± 0.34 *vs* PD = 3.59 ± 0.27 Control vs PD-CI = 3.85 ± 0.43 **60** **min;** ***p*** = **n.s**. Control = 3.64 ± 0.42 vs PD = 3.51 ± 0.28 Control vs PD-CI = 3.44 ± 0.36 **120** **min;** ***p*** = **n.s**. Control = 3.87 ± 0.52 vs PD = 3.67 ± 0.34 Control vs PD-CI = 3.82 ± 0.43 **180** **min;** ***p*** = **n.s**. Control = 4.21 ± 0.54 vs PD = 3.92 ± 0.35Control vs PD-CI = 4.46 ± 0.43
Majeed; Al-Lami; AlGawwam^39^	NA	NA

*PD* Parkinson Disease, *PD-CI* PD with cognitive impairment, *SD* Standard deviation, *SEM* Standard error of the mean, *95% CI* Confidence interval, *Q1* First quartile is the 25th percentile of the data set, *Q3* Third quartile is the 75th percentile of the data set, *EMM* Estimated marginal means, *SE* Standard error, *NA* Not assessed.

^a^The data was transformed by estimation into mean ± SD for the meta-analysis.

### Data analysis

We assessed the risk of bias in the quasi-experimental studies using the tool developed by the Joanna Briggs Institute (JBI)^73^; we assessed cross-sectional studies using the tool proposed by the Specialist Unit for Review Evidence (SURE). Both evaluations were carried out independently by two authors (Gouveia, H. J. C. B. and Santos-Junior, O. H.).

We performed a meta-analysis of continuous data on fasting and postprandial plasma ghrelin concentrations in individuals with PD and controls. We extracted the data using WebPlotDigitizer 5.2 (Automeris LLC, California, USA <plots@automeris.io > ) from the studies that presented graphics rather than tables (Table S3). We estimated the data from the studies that were not presented in mean and standard deviation according to Luo et al. (2018)^74^ and Wan et al. (2014)^75^ (Tables S4 and S5). We did not include a study in the analysis of fasting and postprandial ghrelin due to the absence of mean and standard deviation data and the unfeasibility of estimating them^36^. The authors did not respond to our requests for data in the required format. We included another with incomplete data for the postprandial analysis (only 180 min) due to the absence of a reply from the authors to obtain complete data^35^. Considering the possible differences between total and active ghrelin concentrations, we analyzed these variables and divided them into subgroups. Due to the heterogeneity of the postprandial analyses, we have compiled the results obtained without subdividing them by the evaluation time (in minutes) after the intervention (meal). The effects were expressed as standard mean difference (SMD). We presented the pooled data with a 95% confidence interval (CI) and weighted these results using a random effects model. We assessed the heterogeneity between studies using the I^2^ index, with values between 30% and 60% indicating moderate heterogeneity and values above 75% indicating considerable heterogeneity. We analyzed the probability of publication bias using funnel plots and prepared the review according to PRISMA (Preferred Reporting Items for Systematic Reviews and Meta-Analyses) statement (Table S6). To run all the data, we use the Review Manager (RevMan, Version 5.4, The Cochrane Collaboration, 2020).

## Supplementary information

Supplementary information

## Data Availability

All data supporting the findings of this study are available within the paper and its Supplementary Information.
