# Supplementary material for: Fasting and postprandial Ghrelin levels in Parkinson’s disease: a systematic review and meta-analysis
Source: NPJ Parkinsons Dis. 2025 Jul 14;11:212. doi: 10.1038/s41531-025-01066-0 (PMC12259941; doi:10.1038/s41531-025-01066-0)
Supplement: Supplementary file 1 — Supplementary information [file 41531_2025_1066_MOESM1_ESM.pdf]

Table S1. Risk of bias analysis of quasi-experimental studies.

	INTERNAL VALIDITY BIAS RELATED TO:									
**Study**	**Temporal precedence (1)**	**Selection and allocation (2)**	**Confounding factors (3)**	**Administration of intervention/exposure (4)**	**Assessment, detection, and measurement of the outcome (5, 6, and 7)**			**Participant retention (8)**	**Statistical conclusion validity (9)**
**Unger et al. 2011**	Unclear	Yes	Yes	No	Yes	Yes	Yes	Yes	Yes
**Song et al. 2017**	Unclear	Yes	Yes	No	Yes	Yes	Yes	Yes	Unclear
**Tarianyk et al. 2020**	Unclear	Yes	No	Unclear	No	Yes	Yes	Yes	Unclear
**Pietraszko et al. 2020**	Unclear	Yes	Yes	Yes	Yes	Yes	Yes	Yes	Yes
**Hornsby et al. 2020**	Unclear	Yes	Yes	Yes	Yes	Yes	Yes	Yes	Yes
**Siervo et al. 2024**	Unclear	Yes	Yes	No	Yes	Yes	Yes	Yes	Yes

(1) Is it clear in the study what is the "cause" and what is the "effect" (ie, there is no confusion about which variable comes first)?; (2) Was there a control group?; (3) Were the participants included in any comparisons similar?; (4) Were the participants included in any comparisons receiving similar treatment/care, other than the exposure or intervention of interest? (We considered studies that withheld any type of intervention at the time of blood collection to be at low risk of bias); (5) Were there multiple measurements of the outcome both pre and post the intervention/exposure? (whether multiple measurements of the outcome were taken after the "intervention" [meal]); (6) Were the outcomes of participants included in any comparisons measured in the same way?; (7) Were outcomes measured in a reliable way?; (8) Was follow-up complete and, if not, were differences between groups in terms of their follow-up adequately described and analyzed?; (9) Was appropriate statistical analysis used?

Table S2. Risk of bias analysis of cross-sectional studies.

Study	Is the study design clearly stated?	Does the study address a clearly focused question?	Are the setting, locations and relevant dates provided?	Were participants fairly selected?	Are participant characteristics provided?	Are the measures of exposures & outcomes appropriate?	Is there a description of how the study size was arrived at?	Are the statistical methods well described?	Is information provided on participant eligibility?	Are the results well described?	Is any sponsorship/conflict of interest reported?	Did the authors identify any limitations and, if so, are they captured above?
**Majeed et al. 2024**	Yes	Yes	Yes	Yes	Yes	Yes	No	No	Yes	Yes	Yes	No
**Fiszer et al. 2010**	Yes	Yes	No	Yes	Yes	Yes	No	No	Yes	Yes	Yes	No

Table S3. Data extracted from the study graphs.

Song et al. 2017						Siervo et al. 2024					
Fasting total Ghrelin				n	SEM	Total Ghrelin				n	
Male	C	Mean	514.16	159	16.91	C		EMM	6.27	20	
	C	SEM	531.08			C		SE	0.49		
	PD	Mean	453.28	160	11.84	PD		EMM	6.04	18	
	PD	SEM	465.12			PD		SE	0.34		
Female	C	Mean	649.47	144	25.37	PD-CI		EMM	5.96	15	
	C	SEM	674.84			PD-CI		SE	0.43		
	PD	Mean	539.53	131	18.60						
	PD	SEM	558.14								
Fasting active Ghrelin				n	SEM	Active Ghrelin				n	
Male	C	Mean	122.12	159	9.29	C		EMM	4.31	18	
	C	SEM	131.41			C		SE	0.43		
	PD	Mean	81.85	160	5.76	PD		EMM	4.14	18	
	PD	SEM	87.61			PD		SE	0.28		
Female	C	Mean	149.11	144	10.18	PD-CI		EMM	4.35	15	
	C	SEM	159.29			PD-CI		SE	0.35		
	PD	Mean	96.01	131	7.08						
	PD	SEM	103.09								
Postprandial total Ghrelin				n	SEM	Postprandial total Ghrelin				n	SE
Controle	0	Mean	549.35	303	43.97	Controle	0	EMM	-	20	-
		SEM	593.32					SE	-		
	30	Mean	484.28		31.66		5	EMM	6.23		0.43
		SEM	515.94					SE	6.66		
	90	Mean	486.04		26.38		15	EMM	6.19		0.48
		SEM	512.42					SE	6.67		
	180	Mean	628.49		42.21		30	EMM	6.16		0.37
		SEM	670.7					SE	6.53		
PC	0	Mean	403.38	291	29.90		60	EMM	5.95		0.36
		SEM	433.28					SE	6.31		

	30	Mean	377,00		19.35
		SEM	396.35		
	90	Mean	343.59		17.58
		SEM	361.17		
	180	Mean	401.62		24.62
		SEM	426.24		
Postprandial active Ghrelin				n	SEM
Controle	0	Mean	118.79	303	14.12
		SEM	132.91		
	30	Mean	87.01		10.59
		SEM	97.6		
	90	Mean	111.72		10.60
		SEM	122.32		
	180	Mean	255.59		24.71
		SEM	280.3		
PC	0	Mean	127.61	291	22.95
		SEM	150.56		
	30	Mean	89.66		11.47
		SEM	101.13		
	90	Mean	72.89		10.59
		SEM	83.48		
	180	Mean	115.25		15.89
		SEM	131.14		

	120	EMM	6.02		0.38
		SE	6.4		
	180	EMM	6.22		0.40
		SE	6.62		
PD	0	EMM	-	18	-
		SE	-		
	5	EMM	5.98		-0.30
		SE	5.68		
	15	EMM	5.92		0.35
		SE	6.27		
	30	EMM	5.87		0.25
		SE	6.12		
	60	EMM	5.81		0.25
		SE	6.06		
	120	EMM	5.7		0.26
		SE	5.96		
	180	EMM	5.81		0.28
		SE	6.09		
PD-CI	0	EMM	-	15	-
		SE	-		
	5	EMM	6.67		-0.77
		SE	5.9		
	15	EMM	5.86		0.40
		SE	6.26		
	30	EMM	5.96		0.32
		SE	6.28		
	60	EMM	5.88		0.31
		SE	6.19		
	120	EMM	5.92		0.33
		SE	6.25		
	180	EMM	6.12		0.35
		SE	6.47		
Postprandial active Ghrelin				n	SE
Control	0	EMM	-	20	-
		SE	-		

	5	EMM	4.19		0.44
		SE	4.63		
	15	EMM	4.19		0.45
		SE	4.64		
	30	EMM	3.55		-0.34
		SE	3.21		
	60	EMM	3.64		0.42
		SE	4.06		
	120	EMM	3.87		0.52
		SE	4.39		
	180	EMM	4.21		0.54
		SE	4.75		
PD	0	EMM	-	18	-
		SE	-		
	5	EMM	3.99		0.29
		SE	4.28		
	15	EMM	3.83		0.29
		SE	4.12		
	30	EMM	3.59		-0.27
		SE	3.32		
	60	EMM	3.51		0.28
		SE	3.79		
	120	EMM	3.67		0.34
		SE	4.01		
	180	EMM	3.92		0.35
		SE	4.27		
PD-CI	0	EMM	-	15	-
		SE	-		
	5	EMM	4.02		0.36
		SE	4.38		
	15	EMM	3.57		0.35
		SE	3.92		
	30	EMM	3.85		0.43
		SE	4.28		
	60	EMM	3.44		-0.36

		SE	3.08		
	120	EMM	3.82		0.43
		SE	4.25		
	180	EMM	4.46		0.43
		SE	4.89		

Table S4. Estimation of sample mean and standard deviation for meta-analysis (fasting ghrelin)

Authors	Fasting ghrelin (mean ± SD)	Method
**Tarianyk et al. 2020**	**Plasma total ghrelin serum concentrations (pg/ml)**<br>Control = 1495.86 ± 287.38<br>Mixed PD = 1305.49 ± 303.29<br>Akinetic-rigid PD = 1156.12 ± 418.37	Estimated mean of the sample from Luo et al. (2018)<br>Estimated standard deviation of the sample from Wan et al. (2014)<br><br>D. Luo, X. Wan, J. Liu and T. Tong (2018), "Optimally estimating the sample mean from the sample size, median, mid-range and/or mid-quartile range", Statistical Methods in Medical Research, 27: 1785-1805.<br>X. Wan, W. Wang, J. Liu and T. Tong (2014), "Estimating the sample mean and standard deviation from the sample size, median, range and/or interquartile range", BMC Medical Research Methodology, 14: 135.
**Song et al. 2017**	**Plasma total ghrelin serum concentrations (pg/ml)**<br>Male<br>Control = 514.16 ± 213.22<br>PD = 453.28 ± 149.76<br>Female<br>Control = 649.47 ± 304.44<br>PD = 539.53 ± 212.88<br>**Plasma active ghrelin concentrations (pg/ml)**<br>Male<br>Control = 122.12 ± 117.14<br>PD = 81.85 ± 72.85<br>Female<br>Control = 149.11 ± 122.16<br>PD = 96.01 ± 81.03	Estimated from GraphPad QuickCalcs<br>Available in: https://www.graphpad.com/quickcalcs/CImean1/?Format=SEM
**Hornsby et al. 2020**	**Plasma total ghrelin serum concentrations (pg/ml)**<br>Control = 541.0 ± 393.45<br>PD = 647.9 ± 486.12<br>PD Dementia = 377.8 ± 217.78<br>**Plasma active ghrelin concentrations (pg/ml)**<br>Control = 78.93 ± 61.58<br>PD = 114.4 ± 84.16<br>PD Dementia = 51.39 ± 48.76	Estimated from GraphPad QuickCalcs<br>Available in: https://www.graphpad.com/quickcalcs/CImean1/?Format=SEM

Table S5. Estimation of sample mean and standard deviation for meta-analysis (postprandial ghrelin)

Authors	Total ghrelin (mean ± SD)	Active ghrelin (mean ± SD)	Method
**Tarianyk et al. 2020**	**120 min (pg/ml)**<br>Control = 1333.01 ± 297.36<br>Mixed PD = 1326.84 ± 231.25<br>Akinetic-rigid PD = 1101.47 ± 403.24	NA	Estimated mean of the sample from Luo et al. (2018)<br>Estimated standard deviation of the sample from Wan et al. (2014)<br><br>D. Luo, X. Wan, J. Liu and T. Tong (2018), "Optimally estimating the sample mean from the sample size, median, mid-range and/or mid-quartile range", Statistical Methods in Medical Research, 27: 1785-1805.<br>X. Wan, W. Wang, J. Liu and T. Tong (2014), "Estimating the sample mean and standard deviation from the sample size, median, range and/or interquartile range", BMC Medical Research Methodology, 14: 135.
**Song et al. 2017**	**0 min (pg/ml)**<br>Control = 549.35 ± 196.63<br>PD = 403.38 ± 133.71<br>**30 min (pg/ml)**<br>Control = 484.28 ± 141.58<br>PD = 377.00 ± 86.53<br>**90 min (pg/ml)**<br>Control = 486.04 ± 117.97<br>PD = 343.59 ± 78.62<br>**180 min (pg/ml)**<br>Control = 628.49 ± 188.76<br>PD = 401.62 ± 110.10	**0 min (pg/ml)**<br>Control = 118.79 ± 63.14<br>PD = 127.61 ± 102.63<br>**30 min (pg/ml)**<br>Control = 87.01 ± 47.35<br>PD = 89.66 ± 51.29<br>**90 min (pg/ml)**<br>Control = 111.72 ± 47.40<br>PD = 72.89 ± 47.35<br>**180 min (pg/ml)**<br>Control = 255.59 ± 110.50<br>PD = 115.25 ± 71.06	Estimated from GraphPad QuickCalcs<br>Available in:<br>https://www.graphpad.com/quickcalcs/CImean1/?Format=SEM
**Hornsby et al. 2020**	**180 min (pg/ml)**<br>Control = 500.4 ± 65.80<br>PD = 566.1 ± 96.89<br>PD Dementia = 401.8 ± 88.61	**180 min (pg/ml)**<br>Control = 72.86 ± 10.75<br>PD = 112.0 ± 18.31<br>PD Dementia = 41.32 ± 18.25	Estimated from GraphPad QuickCalcs<br>Available in:<br>https://www.graphpad.com/quickcalcs/CImean1/?Format=SEM

Table S6. PRISMA 2020 Checklist.

Section and Topic	Item #	Checklist item	Location where item is reported
**TITLE**			
Title	1	Identify the report as a systematic review.	L. 1-2
**ABSTRACT**			
Abstract	2	See the PRISMA 2020 for Abstracts checklist.	L. 19-31
**INTRODUCTION**			
Rationale	3	Describe the rationale for the review in the context of existing knowledge.	L. 101-112
Objectives	4	Provide an explicit statement of the objective(s) or question(s) the review addresses.	L. 105-109
**METHODS**			
Eligibility criteria	5	Specify the inclusion and exclusion criteria for the review and how studies were grouped for the syntheses.	L. 365-372
Information sources	6	Specify all databases, registers, websites, organisations, reference lists and other sources searched or consulted to identify studies. Specify the date when each source was last searched or consulted.	L. 354-356
Search strategy	7	Present the full search strategies for all databases, registers and websites, including any filters and limits used.	L. 358-360
Selection process	8	Specify the methods used to decide whether a study met the inclusion criteria of the review, including how many reviewers screened each record and each report retrieved, whether they worked independently, and if applicable, details of automation tools used in the process.	L. 363-374
Data collection process	9	Specify the methods used to collect data from reports, including how many reviewers collected data from each report, whether they worked independently, any processes for obtaining or confirming data from study investigators, and if applicable, details of automation tools used in the process.	L. 375-383
Data items	10a	List and define all outcomes for which data were sought. Specify whether all results that were compatible with each outcome domain in each study were sought (e.g. for all measures, time points, analyses), and if not, the methods used to decide which results to collect.	L. 379-383
	10b	List and define all other variables for which data were sought (e.g. participant and intervention characteristics, funding sources). Describe any assumptions made about any missing or unclear information.	L. 376-378
Study risk of bias assessment	11	Specify the methods used to assess risk of bias in the included studies, including details of the tool(s) used, how many reviewers assessed each study and whether they worked independently, and if applicable, details of automation tools used in the process.	L. 384-388
Effect measures	12	Specify for each outcome the effect measure(s) (e.g. risk ratio, mean difference) used in the synthesis or presentation of results.	L. 403
Synthesis methods	13a	Describe the processes used to decide which studies were eligible for each synthesis (e.g. tabulating the study intervention characteristics and comparing against the planned groups for each synthesis (item #5)).	L. 397-399
	13b	Describe any methods required to prepare the data for presentation or synthesis, such as handling of missing summary statistics, or data conversions.	L. 392-396
	13c	Describe any methods used to tabulate or visually display results of individual studies and syntheses.	L. 403-405
	13d	Describe any methods used to synthesize results and provide a rationale for the choice(s). If meta-analysis was performed, describe the model(s), method(s) to identify the presence and extent of statistical heterogeneity, and software package(s) used.	L. 403-411
	13e	Describe any methods used to explore possible causes of heterogeneity among study results (e.g. subgroup analysis, meta-regression).	L. 405-407
	13f	Describe any sensitivity analyses conducted to assess robustness of the synthesized results.	L. 399-403
Reporting bias assessment	14	Describe any methods used to assess risk of bias due to missing results in a synthesis (arising from reporting biases).	L. 407-409
Certainty assessment	15	Describe any methods used to assess certainty (or confidence) in the body of evidence for an outcome.	L. 403-410
**RESULTS**			

Section and Topic	Item #	Checklist item	Location where item is reported
Study selection	16a	Describe the results of the search and selection process, from the number of records identified in the search to the number of studies included in the review, ideally using a flow diagram.	Fig. 1
	16b	Cite studies that might appear to meet the inclusion criteria, but which were excluded, and explain why they were excluded.	Fig. 1
Study characteristics	17	Cite each included study and present its characteristics.	L. 163-175
Risk of bias in studies	18	Present assessments of risk of bias for each included study.	L. 122-162
Results of individual studies	19	For all outcomes, present, for each study: (a) summary statistics for each group (where appropriate) and (b) an effect estimate and its precision (e.g. confidence/credible interval), ideally using structured tables or plots.	Table 2 and 3<br>Fig. 2 and 5
Results of syntheses	20a	For each synthesis, briefly summarise the characteristics and risk of bias among contributing studies.	L. 217-219 (Fig. 3 and 4)<br>L. 227-228 (Fig 6 and 7)
	20b	Present results of all statistical syntheses conducted. If meta-analysis was done, present for each the summary estimate and its precision (e.g. confidence/credible interval) and measures of statistical heterogeneity. If comparing groups, describe the direction of the effect.	L. 210-219<br>L. 220-228
	20c	Present results of all investigations of possible causes of heterogeneity among study results.	L. 213-215<br>L. 223-225
	20d	Present results of all sensitivity analyses conducted to assess the robustness of the synthesized results.	L. 215-217<br>L. 226-227
Reporting biases	21	Present assessments of risk of bias due to missing results (arising from reporting biases) for each synthesis assessed.	L. 217-219<br>L. 227-228
Certainty of evidence	22	Present assessments of certainty (or confidence) in the body of evidence for each outcome assessed.	L. 213-219<br>L. 223-228
**DISCUSSION**			
Discussion	23a	Provide a general interpretation of the results in the context of other evidence.	L. 230-233
	23b	Discuss any limitations of the evidence included in the review.	L. 338-343
	23c	Discuss any limitations of the review processes used.	L. 338-343
	23d	Discuss implications of the results for practice, policy, and future research.	L. 344-352
**OTHER INFORMATION**			
Registration and protocol	24a	Provide registration information for the review, including register name and registration number, or state that the review was not registered.	L. 360-362
	24b	Indicate where the review protocol can be accessed, or state that a protocol was not prepared.	L. 360-362
	24c	Describe and explain any amendments to information provided at registration or in the protocol.	L. 360-362
Support	25	Describe sources of financial or non-financial support for the review, and the role of the funders or sponsors in the review.	L. 416-422
Competing interests	26	Declare any competing interests of review authors.	L. 430
Availability of data, code and other materials	27	Report which of the following are publicly available and where they can be found: template data collection forms; data extracted from included studies; data used for all analyses; analytic code; any other materials used in the review.	L. 413-414

From: Page MJ, McKenzie JE, Bossuyt PM, Boutron I, Hoffmann TC, Mulrow CD, et al. The PRISMA 2020 statement: an updated guideline for reporting systematic reviews. BMJ 2021;372:n71. doi: 10.1136/bmj.n71. This work is licensed under CC BY 4.0. To view a copy of this license, visit https://creativecommons.org/licenses/by/4.0/
